# An informative short-term study on the impacts of a triclocarban/weathered multi-walled carbon nanotube-adsorbed complex to benthic organisms

**DOI:** 10.1007/s11356-024-32447-2

**Published:** 2024-02-17

**Authors:** Katrin Weise, Stephan Beil, Klemens Schwanebeck, Alina Catrinel Ion, Thomas Ulrich Berendonk, Dirk Jungmann

**Affiliations:** 1 Faculty of Environmental Sciences, Institute of Hydrobiology, Technische Universität Dresden, Zellescher Weg 40, 01217 Dresden, Germany; 2 Faculty of Environmental Sciences, Institute of Water Chemistry, Technische Universität Dresden, Bergstraße 66, 01062 Dresden, Germany; 3 Faculty of Chemical Engineering and Biotechnologies, National University of Science and Technology Politehnica Bucharest, 1-7 Gheorghe Polizu St., Sector 1, 011061 Bucharest, Romania

**Keywords:** Weathered multi-walled carbon nanotubes, Adsorption, Benthic biofilm, *L. stagnalis*, Triclocarban

## Abstract

Freshwater organisms are suitable models to study the fate of environmental pollutants. Due to their versatile and everyday use, many environmental pollutants such as triclocarban (TCC) or multi-walled carbon nanotubes (MWCNTs) enter environmental compartments very easily. TCC is known as a disinfectant and is declared as a highly aquatic toxicant. Multi-walled carbon nanotubes are used, e.g., in the automotive industry to improve plastic properties. Both TCCs and MWCNTs can pose major pollution hazards to various organisms. In addition, these substances can bind to each other due to their tendency to interact via strong hydrophobic interactions. Therefore, a short-term test was conducted to investigate the effects of the individual chemicals TCC and weathered MWCNTs (wMWCNTs) on a benthic biofilm and a grazing organism, *Lymnaea stagnalis*. Furthermore, the two compounds were coupled by an adsorption experiment resulting in a coupled complex formation (TCC + wMWCNTs). *L. stagnalis* showed no effects in terms of mortality. For benthic biofilm, the coupling test (TCC + wMWCNTs) showed a decrease of 58% in chlorophyll *a* (Chl-*a*) concentration. The main effect could be attributed to the wMWCNTs’ exposure alone (decrease of 82%), but not to presence of TCC. The concentration range of Chl-*a* upon TCC exposure alone was comparable to that in the control group (32 and 37 µg/cm^2^). With respect to the particulate organic carbon (POC) concentration, very similar results were found for the solvent control, the TCC, and also for the TCC + wMWCNTs group (3, 2.9, and 2.9 mg/cm^2^). In contrast to the control, a significant increase in POC concentration (100%) was observed for wMWCNTs, but no synergistic effect of TCC + wMWCNTs was detected.

## Introduction

As described several times in the literature, triclocarban (TCC) is among the top 10 contaminants of concern (CEC) in terms of abundance and concentration in the environment (Lu et al. 2019; Kolpin et al. 2002; Halden and Paull 2005). In fact, TCC has been detected more frequently and at higher concentrations in wastewater treatment plant effluents and surface waters than triclosan (TCS), a fairly similar disinfectant (Coogan et al. 2007). To date, few studies have been conducted on the mode of toxic action of TCC, but it is nevertheless classified as extremely hazardous to water. In addition, conflicting measured and estimated values for the water solubility of TCC have been reported, complicating its classification as hazardous to water even further (Tamura et al. 2012; Delgado et al. 2012; Giri et al. 2011; Yun et al. 2020; Wang et al. 2022).

TCC is an anilide and a chemical compound, which is used as an antimicrobial agent in soaps, skin creams, deodorants, and even plastics and is found in surface waters up to 6.75 µg/L (Halden and Paull 2005). In this study, Brausch and Rand (2011) even state a range of measured TCC concentrations between 19 and 1425 ng/L in surface waters. Brausch and Rand (2011) describe that only few aquatic toxicity data are available for TCC compared to TCS, another substance which is applied in the applications mentioned above (Brausch and Rand 2011). Furthermore, they describe TCC as slightly more toxic than TCS toward aquatic invertebrates and fish for both acute and chronic exposure. Based on the most sensitive endpoint studied to date and the highest observed environmental concentration, which was the lowest observed effect concentration (LOEC) for invertebrates in a reproductive test, a hazard quotient of 10.96 was determined for TCC (Brausch and Rand 2011). In contrast, TCS was only found to have hazard quotients ranging from 0.038 based on EC25 (growth test for invertebrates) to 0.230 as LOEC (plant morphology). However, a hazard quotient of 19.167 for TCS was found in a growth inhibition test for algae (LOEC) (Brausch and Rand 2011).

Near wastewater treatment plant outfalls, bioaccumulation factors of 1600 for snails and 2700 for algae can be achieved for TCC (Coogan and La Point 2008; Coogan et al. 2007). For *Daphnia magna*, an LC50 was described at 0.01 mg/L TCC after 48 h in an acute test. The green alga *Scenedesmus subspicatus* showed an LC50 at 0.02 mg/L TCC after 72 h in a growth inhibition test. In *Ceriodaphnia dubia*, however, the LC50 was as low as 0.0031 mg/L TCC after 48 h (Brausch and Rand 2011). Because TCC has a very pronounced tendency for adsorption and benthic biofilms are generally described as sinks for contaminants (see Weise et al. 2021), we tested the effects of TCC on benthic biofilms and the grazing organism *L. stagnalis*. Grazing of benthic biofilms by freshwater snails promotes nutrient turnover in algal communities. These algal compartments may contain antimicrobial agents such as TCC that are incompletely removed during wastewater treatment, resulting in their release into the aquatic environment. Furthermore, we tested weathered multi-walled carbon nanotubes (wMWCNTs). The reason for this is that both substances occur in environmental compartments and have already been detected (Mueller and Nowack 2008; Petersen et al. 2016; Ion et al. 2019). This type of CNTs has been detected in a range of 0.001–0.8 ng/L in surface waters, whereas concentrations between 3.69 and 32.66 ng/L are described for wastewaters, but environmental concentrations of nanomaterials in general are widely unknown (Maurer-Jones et al. 2013; Lawrence et al. 2016). However, there are studies on investigated CNT concentrations in sediments that range between 1 µg/kg and 1 mg/kg (Selck et al. 2016). The structure of CNTs is described by Gusain et al. (2020) as having excellent intrinsic properties. These include a large active surface area, high mechanical and chemical stability as well as high electrical conductivity. These properties show an immense application in electrical and sensor technology. However, these properties are also particularly important for biomedical applications and for adsorption (Gusain et al. 2020). As a result, they are often used for the adsorption of pollutants as well as for the adsorption of water impurities (Upadhyayula et al. 2009; Ihsanullah et al. 2016).

Due to their high sorption properties, TCC and wMWCNTs could serve as vehicles and thus there could be the possibility that TCC becomes more effective (additive effect) (Ion et al. 2021). Depending on temperature, pH, or redox processes in the environment, CNTs in general can adsorb hydrophobic environmental chemicals, which can result in a different accumulation behavior in the environment for both the sorbent and the sorbate. Thus, MWCNTs enter the environment via direct inputs, such as from industrial production, or indirectly via wastewater and landfill leachate (Petersen et al. 2011). TCC and some organics (EPS as well as cellular transport proteins) are able to bind and form some complexes that affect the function of sludge microorganisms. This leads to some possible associations of TCC with other pollutants (Ion et al. 2019; Wang et al. 2023). It is also described that carbon nanotubes have applications in environmental remediation due to its excellent mechanical and chemical properties. Thus, better results are achieved in the removal of organic and inorganic pollutants (Chung et al. 2022). Zhang et al. (2020) describe a study on the degradation of pollutants by carbon nanotubes. Here, it is explained that the presence of CNTs might influence the biodegradation of pollutants and environmental matrices. This depends, among other things, on the properties of the carbon nanotubes, the physicochemical properties of pollutants, and the environmental conditions. This makes it complicated to describe carbon nanotubes in terms of biodegradation (Zhang et al. 2020).

The influences of the chemical composition of the aquatic environment and of the temperature on triclocarban (TCC) sorption on pristine and irradiated multi-walled carbon nanotubes (MWCNTs) at different temperatures were also studied (Ivan et al. 2021, 2023). Natural waters have been characterized in terms of the concentrations of cations and anions, pH, and electric conductivity, the sorption process of TCC on MWCNTs being influenced by the variation of TCC solubility, in aqueous media with different compositions and at different temperatures.

The effects of TCC, a highly used antimicrobial agent with high river sediment contamination, on the benthic community, especially the grazing organism *Lymnaea stagnalis*, have not been reported. However, it is important to investigate a variety of aspects, such as comparing different organisms to each other based on their different uptake pathways. Thus, a better assessment and estimation of the hazard of TCC or a complex of TCC and nanotubes can be ensured. Therefore, we designed a test study with TCC, but also with wMWCNTs as single compounds and the complex of TCC and wMWCNTs (TCC + wMWCNTs). Thus, the effects on a benthic biofilm community, which is much more abundant in nature than single algal species, can be studied. Since there are studies in the literature in this regard, albeit more on TCS, we aim to present a different environmental scenario here. The grazing organism *L. stagnalis* is then used to graze the biofilm in the aquatic environment and was therefore used in our studies.

## Materials and methods

### Microcosm

The microcosm for both benthic biofilm and *L. stagnalis* took place in aquaria (20 × 15 × 18 cm) at an air-conditioned laboratory each for 1 week (5 days). The temperature was kept constant at 20 ± 1 °C with a light/dark cycle of 12:12 h. The benthic biofilm was sampled in the Gauernitzbach and prepared for the experimental setup, which is described in detail by Weise et al. (2021). For the biofilm test, each aquarium was equipped with four glass slides, so that the subsequent analysis could be performed accurately. Living individuals of *L. stagnalis* were obtained from the breeding station INRA (French National Institute for Agricultural Research, France).

### Triclocarban

The applied test substance triclocarban (TCC, CAS No: 101–20-2), 3,4,4′-trichlorodiphenylurea, 1-(4-chlorophenyl)-3-(3,4-dichlorophenyl)-urea, was obtained from Sigma Aldrich. As stock solution, 10 mg TCC was dissolved in 10 mg ethanol (≤ 99.8%; Carl Roth). Subsequently, 5 mL of this stock solution was filled up to 500 mL with distilled water in a volumetric flask (dosing solution). Then, 40 mL of the dosing solution was added to the aquaria containing 360 mL Borgmann medium (Borgmann 1996), resulting in a final volume of 400 mL in all exposure aquaria, and the same volume is given for control and solvent control. In contrast to prior investigations, the TCC identified in the article could be uniformly dispersed in aqueous solution at a concentration of 1 mg/L. TCC is a hydrophobic compound, and trustworthy data on its solubility range from 68 to 114 μg/L (Gaviria-Castillo et al. 2019). The final total concentration of distributed TCC was 1 mg/L. The concentration of 1 mg/L TCC was used for all experiments in the aquaria because different pre-tests with higher concentrations formed a precipitate and thus could not be used. According to LC–MS/MS measurements, the TCC was homogeneously distributed within the aqueous solution at 1 mg/L (initial concentration for TCC: 1055.7 ± 58.0 µg/L). However, we did not prove that TCC exists at this concentration freely dissolved. It could also be finely dispersed. Since there was no mortality of *L. stagnalis*, only this concentration was used.

### Weathered multi-walled carbon nanotubes

The MWCNTs (Baytubes C 150 P; BTS, Leverkusen, Germany) purchased from Bayer MaterialScience AG in 2007 were synthesized. The weathering process of the MWCNTs used here was carried out in a Sunset CPS + device (ATLAS Materials Testing Solutions) using ultraviolet radiation (65 W/m^2^ = 504,440 kJ/m^2^). The wMWCNTs have a length of 1–10 µm and an inner diameter distribution of 5–20 nm. More precise information is described in Politowski et al. (2021). In brief, the following protocol was observed according to ISO 3892–2:2006: the device provided light with a daylight UV filter and a wavelength from 300 to 400 nm and also with an integrated air-cooled xenon lamp (1500 W). The internal sample table was cooled with a constant flow of cold water during this process. The position of sample bins was changed once a week to achieve a uniform irradiation.

### TCC + wMWCNTs (coupled)

The test design accords to Ion et al. (2019); the mixture toxicity experiment was performed under the same conditions as described above. To achieve a good adsorption of TCC to wMWCNTs, a ratio of 1:100 (TCC/wMWCNTs) had to be chosen (Ion et al. 2019). For this purpose, a stock solution of 20 mg TCC in 20 mL ethanol was prepared and dissolved in a volumetric flask (20 mL) at RT. A dosing solution was then prepared by taking 5 mL of the stock solution and filling it up to 500 mL with distilled water in a volumetric flask (500 mL). From this dosing solution, 80 mL was transferred to a 2-L beaker containing 720 mL Borgmann medium. In addition, 80 mg of wMWCNTs was weighed separately and dispersed in 800 mL Borgmann medium for 30 min at RT. Then, both solutions were mixed and stirred at 1500 rpm for 2 h at RT.

### Quantification of the TCC concentration by LC–MS/MS

TCC concentrations in aqueous samples were determined using a QTRAP 6500^+^ ESI triple quadrupole mass detector (Sciex) equipped with a Shimadzu UHPLC system, comprising two Nexera X2 LC-30AD high-pressure pumps, a Nexera X2 SIL-30AC autosampler, and a CTO-20AC column oven. After 1 week, a subsample of 5 mL was taken out of each aquarium for analyzing the TCC amount. For this, the control, solvent control and wMWCNTs were analyzed, but the samples with TCC were diluted in a ratio of 1:1000. Afterwards, all further steps were done at the institute of water chemistry. Then, 5 mL of the sample was thoroughly mixed with 500 µL of potassium phosphate buffer (pH 7.0, 0.1 M), 100 µL of an aqueous EDTA solution (25 g/L), 350 µL of water (LC/MS grade), and 50 µL of a TCC-d4 solution (100 µg/L in 50 vol.% aqueous methanol), which was used as surrogate standard. The samples were shaken vigorously, and 3 mL of the well-suspended sample was filtrated through a syringe filter (0.2 µm, regenerated cellulose). Subsequently, the used filter was removed from the syringe and eluted with 1 mL of acetonitrile (LC/MS grade). The acetonitrile was removed in gentle nitrogen stream at 40 °C to a remaining volume of 0.1 mL, which was subsequently diluted to 1 mL with water (LC/MS grade) and used for the LC–MS/MS measurement.

An EVO C18 UHPLC column (100 × 2.1 mm I.D., 1.6 µm; Kinetex) was operated at 0.6 mL/min and a constant temperature of 40 °C. Water (LC/MS grade) and acetonitrile (LC/MS) grade, both comprising 0.02% of acetic acid, were used as eluents A and B, respectively. The gradient program started at 5% eluent B, which was kept for 1 min, then rising linearly to 30% eluent B at 1.1 min and to 98% eluent B at 4.1 min (kept constant until 6.3 min). Subsequently, the starting conditions were re-established. TCC was detected in negative ESI mode (ion spray voltage of − 4500 V, source temperature at 300 °C, curtain gas at 40 psi, gas 1 and 2 both at 50 psi, medium level of nitrogen as collision gas) using the following mass transitions for MRM measurements: 313/160 as quantifier; 315/162 as qualifier (for TCC-d4: 317/160).

### Benthic biofilm community

TCC was added at the beginning of the experiment to the biofilm dispersion. All further steps to prepare the biofilm solution are described (Weise et al. 2021). Subsequently, the biofilm solution was added to the aquaria either as a control, with wMWCNTs, or with TCC for 1 week, respectively. Each aquarium was equipped with four microscope slides consisting of lime-natron glass (76 × 26 mm) and a width of 1 mm (Roth, Karlsruhe, Germany). After 1 week, the slides, which were overgrown with the benthic biofilm, were randomly sampled for examination of Chl-*a* and POC concentration.

### Analysis of chlorophyll *a*

After 1 week, all four glass slides from each aquarium were kept out and prepared for the appropriate steps for analyzing. The Chl-*a* concentration was determined according to the method of Wetzel and Likens (2000). Samples were filtered through a 25-mm-diameter glass fiber filter (Sartorius). The filtration step was performed at a relative underinflation of 200 mbar with respect to the atmosphere (Dewitt’s underinflation). Afterwards, the filter was placed in a 1.5-mL Eppendorf tube and frozen at − 18 °C for at least 24 h. After freezing, the filter was removed from the tube and crushed with scissors and 5 mL ethanol (90% buffered with 1 g/L MgCO_3_; Merck) added for homogenization with a mixer (Ultra Turrax T18 basic; IKA), for 2 min at 16,000 rpm. The ethanol concentration of 90% was used to extract the pure Chl-*a* from the biofilm. Afterwards, the extracts were stored in the dark for at least 24 h. In a subsequent filtration step, the supernatant was transferred to a shake flask. The final volume was 25 mL. Fluorescence was measured using a luminescence spectrometer (LS 50B, software: FL WinLab 2.0, excitation wavelength 434 nm, emission wavelength 667 nm; Perkin Elmer). The Chl-*a* concentration was determined using the following equation (Eq. 1) (Rybicki 2014):

1 $${\text{Chl}}-a=\frac{F*\left({R}_{{\text{b}}}-{R}_{{\text{a}}}\right)*\tau }{\tau -1}*\frac{{V}_{{\text{extract}}}*{V}_{{\text{total}}}}{{V}_{{\text{filter}}}*A}$$

where Chl-*a*—chlorophyll *a* (μg/cm^2^); *F*—slope of calibration function; *R*_b_—sample fluorescence; *R*_a_—sample fluorescence after acidification; *V*_extract_—extraction volume (mL); *V*_filter_—sample volume filtered (mL); *V*_total_—total water volume for scraping Aufwuchs from glass slides (mL); *τ*—quotient of standard fluorescence before and after acidification.

### Analysis of particulate organic carbon

The carbon-free filter (MGF 0.45 µm; Sartorius) was dried at 70 °C for about 12 to 24 h before usage. Afterwards, the analysis was done in a carbon analyzer (C-200; Leco, USA). For each glass slide, the amount of carbon was normalized to carbon concentration per area with the following equation (Eq. 2.):

2 $${\text{POC}}=\frac{10 * C * {V}_{{\text{total}}}}{{V}_{{\text{filter}}} * A}$$

where POC—particulate organic carbon (mg/cm^2^); *C*—sample carbon concentration (% g^−1^); *V*_total_—total water volume for scraping Aufwuchs from tile (mL); *V*_filter_—sample volume on the filter (mL); *A*—surface area of the tile (cm^2^).

### Test organism *L. stagnalis*

Before starting the experiment, the organisms were reared in Borgmann medium according to the recipe of LO-4S E + H (Borgmann 1996) and fed 3 to 4 times a week with small pieces of organic cucumber and organic salad (Weise et al. 2021). The exposure and control aquaria were performed for 1 week and five animals were put into each aquarium with four replicates each.

### General experimental setup

**Fig. 1:**
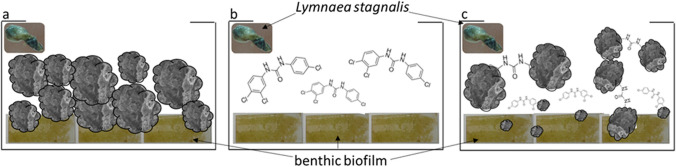
Overview of the experimental setup: **a** microcosm for benthic biofilm on glass slides and *L. stagnalis* concerning wMWCNTs, **b** microcosm for benthic biofilm on glass slides and *L. stagnalis* referring to TCC, **c** microcosm for benthic biofilm on glass slides and *L. stagnalis* regarding TCC + wMWCNTs

The study was performed using the same concentrations in the exposure aquaria, 1 mg/L wMWCNTs as well as 1 mg/L TCC in Fig. 1a and b. Figure c shows the coupled experiment of wMWCNTs + TCC. This design was developed the same for *L. stagnalis* and the benthic biofilm, respectively. The setup was accompanied by reference samples measured at *t*_0_ directly after scraping off, control and solvent control containing ethanol.

### Statistics

Statistics for this experiment were calculated using a one-factor ANOVA with contrast analysis based on treatment contrasts. Significance codes for the test are 0 “***” 0.001 “**” 0.01 “*” 0.05 “.” 0.1 “.” 1.

## Results

### Investigations on the mixture toxicity of TCC to wMWCNTs

**Fig. 2:**
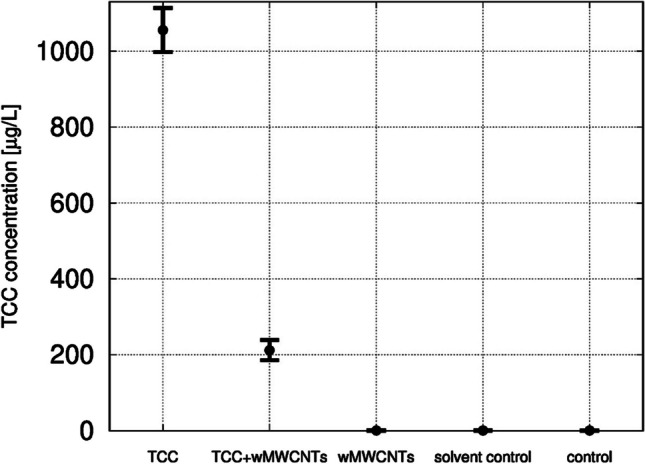
Results of the analysis of TCC (dissolved or well dispersed) via LC/MS at the beginning of the experimental setup depicted as mean ± standard deviation. The analysis of TCC shows a mean of 1055.7 ± 58.0 µg/L. For the coupled complex, an amount of 212.1 ± 26.3 µg/L of TCC was detected. The treatments wMWCNTs, solvent control, and control show amounts of 0.5 ± 0.3 µg/L, 0.5 ± 0.3 µg/L, and 0.4 ± 0.2 µg/L, respectively, *n* = 4

No mortality of snails was observed after 1 week of exposure to TCC, coupled and wMWCNT (non-coupled). Figure 2 shows the total concentration of TCC determined by LC/MS separately and in combination with wMWCNTs at the beginning of the experiment. For this purpose, 4 replicates each were evaluated for TCC, TCC + wMWCNTs, wMWCNTs, solvent control, and control (mean values and standard deviations are listed in Table 1). Based on the TCC concentration of 1056 µg/L, only 20% of the substance could be detected in the supernatant, hence 80% seem to be sorbed to wMWCNTs. Neither in approach with the wMWCNTs nor in the solvent controls or controls, TCC (< 1 µg/L) could be detected. However, the measurement of TCC in the presence of wMWCNTs is negatively affected by the partial sorption of the surrogate standard TCC-d4 to the wMWCNTs. The sorption of the surrogate standard will reduce its signal intensity, what in turn might lead to an overestimation of the remaining TCC concentration after coupling to the wMWCNTs. Therefore, the determined sorption efficiency of 80% should be considered a minimum value. The efficiency might be even higher.

**Table 1:** Overview of the statistics of Chl-*a* with samples, mean, SD as standard deviation and Pr( >|*t*|) represents the *p*-value, which is associated with the *t*-value (reference, REF, are not shown)

Sample	Mean (µg/cm^2^)	SD (µg/cm^2^)	Pr( >|*t*|)
C	32.07	8.52	3.74e − 05***
SC	39.34	14.91	0.3698
TCC	37.25	26.70	0.5199
Coupled	13.36	9.11	0.0311*
wMWCNTs	5.73	3.12	0.0044**

### Chlorophyll *a*

**Fig. 3:**
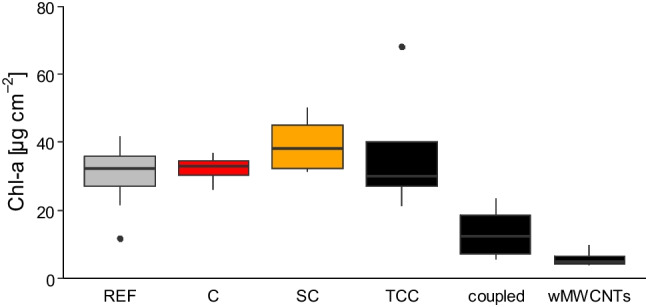
Results of Chl-*a* investigation with mean and relative standard deviation after 5 days (µg/cm.^2^) with reference (gray plotted), control (red plotted), solvent control (SC, yellow plotted), TCC (black plotted), coupled as the complex for TCC and wMWCNTs (black plotted) and wMWCNTs (black plotted), *n* = 4 (statistics are shown in Table 1)

In Fig. 3, the Chl-*a* concentrations are depicted. The reference (mean 30.2 ± 9.6 µg/cm^2^), the controls (mean 32.1 ± 8.5 µg/cm^2^, see Table 1), and solvent control groups (mean 39.3 ± 14.9 µg/cm^2^) are not significantly different, but show a similar trend in Chl-*a* concentration. Furthermore, this is true for the benthic biofilm group, which falls within the ranges. However, a significant decrease in Chl-*a* concentration at 1 mg/L of wMWCNTs (mean 5.7 ± 3.1 µg/cm^2^) can be seen. Thus, a greater influence by wMWCNTs is recognizable than for TCC (mean 37.3 ± 26.7 µg/cm^2^). In the case of the TCC + wMWCNTs coupled, we were able to demonstrate that this group behaves differently. It lies exactly between the TCC-only group and the wMWCNT-only group with a mean and standard deviation of 13.4 ± 9.1 µg/cm^2^. This clearly shows that the TCC + wMWCNTs complex is not additive, but has a mitigated effect for both TCC and wMWCNTs, with the effect clearly in favor of the wMWCNTs.

### Particulate organic carbon

**Fig. 4:**
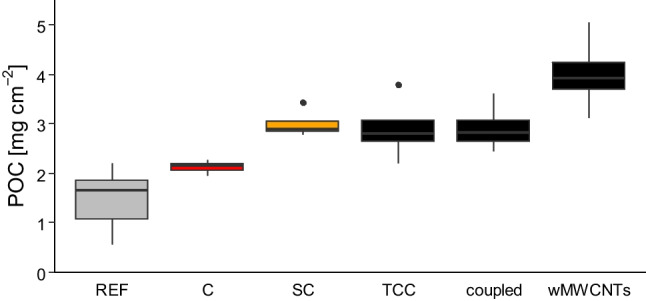
Results of POC investigation with mean and relative standard deviation after 5 days (mg/cm.^2^) with reference (gray plotted), control (red plotted), solvent control (SC, yellow plotted), TCC (black plotted), coupled as the complex for TCC and wMWCNTs (black plotted), and wMWCNTs (black plotted), *n* = 4 (statistics are shown in Table 2)

The POC concentration (Fig. 4) in the reference group (mean 1.5 ± 0.6 mg/cm^2^, see Table 2) compared to the control (mean 2.1 ± 0.1 mg/cm^2^) shows no significant differences, but a slight increase in the control group is discernible. In addition, no different trends in the solvent control (mean 3.0 ± 0.3 mg/cm^2^) and TCC group (2.9 ± 0.7 mg/cm^2^) are analyzed. Treatment with wMWCNTs reveals significant differences from the control group in terms of POC concentration (mean 4.0 ± 0.8 mg/cm^2^). With regard to TCC + wMWCNTs, a similar plot trend as TCC is observed (mean 2.9 ± 0.5 mg/cm^2^). Here again, as with the Chl-*a* concentration, the largest effect can be attributed to the wMWCNTs.

**Table 2:** Overview of the statistics of POC with samples, mean, SD as standard deviation and Pr( >|*t*|) represents the *p*-value, which is associated with the *t*-value (reference, REF, are not shown)

Sample	Mean (mg/cm^2^)	SD (mg/cm^2^)	Pr( >|*t*|)
C	2.12	0.14	8.78e − 07***
SC	2.99	0.29	0.034390*
TCC	2.89	0.66	0.057581
Coupled	2.92	0.49	0.051197
wMWCNTs	4.00	0.79	0.000154***

## Discussion

The results of our study show no mortality in the acute toxicity tests of TCC, wMWCNTs, and the coupled complex TCC + wMWCNTs toward *Lymnaea stagnalis* at any of the investigated concentrations. The benthic biofilm control group showed no significant differences compared to the reference group. In summary, there were no adverse effects from the experimental test conditions.

In the literature, a lot of data based on experiments with single species is available, often referring to single algae, as well as to daphnia or snails as higher organisms. Coogan and La Point (2008) studied the bioaccumulation of TCC and other disinfectants in the snail *Helisoma trivolvis* and the alga *Cladophora* spp. in a stream polluted by sewage treatment plant effluent (Coogan et al. 2007). After 2 weeks of adaption, the snails were collected along with water column samples from the outlet of the wastewater treatment plant and analyzed for TCC by liquid chromatography–mass spectrometry. TCC concentrations in the water samples were low (40–200 ng/L). TCC was elevated to low ppb concentrations (50–300 ng/g fresh weight) in snail samples and also to low ppb concentrations (50–400 ng/g fresh weight) in algal samples. The resulting BAFs for snails and algae were approximately in the range of 10^3^. Lu et al (2019) investigated the effects of TCC on three different single algal species in a 96-h culture exposure test with final concentrations of TCC of 0.5, 1, 2, 4, 8, and 16 mg/L (Lu et al. 2019). They used a growth inhibition test of algae and calculated the 96‑h median effective concentration (EC_50_). The results showed a significant decrease of the Chl-*a* concentration, which was described to be even greater than the effect on the biomass growth. The authors argued that there is a selective toxic effect of TCC on photosynthetic systems in algae. Contrary to this, Tamura et al. (2012) reported about the effects of TCC toward the green alga *Pseudokirchneriella subcapitata* (Tamura et al. 2012). Deploying a growth inhibition test, only 29 µg/L (0.029 mg/L) was found to be the acute toxicity value. For *D. magna*, an EC_50_ value of 10 µg/L (0.010 mg/L) TCC was found. For *Oryzias latipes*, a LC_50_ value of 85 µg/L (0.085 mg/L) was reported. Here, it becomes clear that different results are obtained with the very similar individual algae species (green algae). For snails, Geiß et al. (2016) and Coogan and La Point (2008) analyzed certain toxic effects (Geiß et al. 2016; Coogan and La Point 2008). In the study by Geiß et al. (2016), the chronic effects of TCC on reproduction were investigated using a reproduction test in the mud snail *Potamopyrgus antipodarum*. Mollusks were exposed to concentrations ranging from 0.1 to 10 µg/L TCC for 28 days. The snails were then dissected and the embryos in the brood pouch were counted so that the individual reproductive success of the adult snails could be evaluated. The NOEC and the LOEC were 0.082 µg/L and 0.287 µg/L, respectively. *D. magna* are filter feeders that continuously ingest liquid surrounding medium (Geiß et al. 2016). It has been proven that substances appear in their intestine very quickly after ingestion. Lotocka (2001) reported that the intestine fills within 30 min after ingestion of food under optimal conditions (Lotocka 2001). *L. stagnalis*, on the other hand, is a grazing organism, which takes up food with its radula. Mollusks are in the water and are thus fully exposed to the TCC solution but can probably ingest less solution due to their radula. Concentrations of 6.75 ng TCC/L have been measured in surface waters (Miller et al. 2010). However, considering the low water solubility of TCC with its high adsorption potential, the question is rather whether the most important TCC exposure pathway really occurs via direct contact to surface water or if the benthic exposure should receive more attention. In the latter case, the benthos might be of utmost importance since TCC adsorbs and is then more likely to be found at the organic material. According to our results, TCC was removed from the water phase within a short period in the exposure aquaria. Taking the corresponding non-effects of the benthic organisms into account, it is rather reasonable to assume that a major part of the TCC rapidly adsorbed to the glass wall of the aquarium. Further research should focus on this issue since we could demonstrate that it the adsorption might influence the relevance of the uptake pathways of the respective organisms. In the case of *D. magna*, many studies (mentioned above) have shown effects already after a short time and with different amounts of TCC. However, filter animals are able to absorb the applied TCC concentration in full extent right at the beginning, before the TCC can adsorb to the exposure vessel. The results of the benthic biofilm investigations in the present study, if compared to other studies with single algal species, allow to conclude that community conditions may play a key role. Individual algal species are very likely more sensitive to various impacts of TCC than the communities, which were considered in the present study (Vimalkumar et al. 2019; Lu et al. 2019; Brausch and Rand 2011). Therefore, the 72 h or 96 h of exposure commonly applied in other tests with individual algal species cannot explain the difference (e.g., Brausch and Rand 2011). *L. stagnalis* also appears to tolerate TCC better over the 5-day period than *Daphnia* within the 48-h test applied (Brausch and Rand 2011). The different results for the different organisms mentioned suggest that the different uptake pathways of the respective benthic organisms may be responsible. Individual cells may be more susceptible to stress than has been observed for the benthic community. Contrary to the study of Simon et al. (2014), TCC did not lead to significant effects toward *L. stagnalis* and the benthic biofilm community (Simon et al. 2014).

Concerning the effects of the wMWCNT as the intended carrier, it can be seen that wMWCNTs tested individually are responsible for the major effects and lead to significant reduction of Chl-*a* and POC concentrations. A risk estimate for wMWCNTs has already been determined (Weise et al. 2021). Considering the MEC of 1 mg/kg sediment, a risk quotient of 0.1 was calculated (Selck et al. 2016). However, these calculations suggest that the risk quotient for sediment compartments is thousands of times higher than for the water phase. These calculations underscore the effects identified here. The ability of carbon nanotubes to act as adsorbents is due to their specific physical and surface functional properties (Upadhyayula et al. 2009). However, the adsorption kinetics between organic compounds and CNTs in general have been studied only in recent years (Oleszczuk et al. 2009; Arasteh et al. 2010; Chen et al. 2009; Shen et al. 2009; Sheng et al. 2010).

The adsorption mechanisms between TCC and wMWCNTs involve non-covalent forces such as hydrogen bonding, van der Waals forces, π–π bonds, hydrophobic interactions, and electrostatic forces (Gupta and Saleh 2013; Simon et al. 2015). MWCNTs generally have a large surface area and exhibit strong hydrophobicity (Chen et al. 2007). TCC has a *n*-octanol/water partition coefficient (log *K*_ow_) of 4.9; thus, the hydrophobic effect can be considered the most important factor for the adsorption of TCC on wMWCNTs (Ying et al. 2007). Ilyas et al. (2021) explained that the physicochemical properties of the resulting organic contaminants, i.e., water solubility (WS), octanol–water partition coefficient (log *K*_ow_), or soil organic carbon sorption coefficient (log *K*_oc_), play a very important role in treatment processes. In the present experiment, 80% of TCC could be adsorbed to wMWCNTs, resulting in a coupled TCC/wMWCNT complex. It was observed that the adsorption capacity on irradiated MWCNTs decreases compared to non-irradiated MWCNTs (Ion et al. 2019). It has also been mentioned in the literature that increasing the number of oxygen-containing functional groups on the surface of the nanostructures reduces their adsorption capacity for hydrophobic impurities (Zhou et al. 2013).

This complex showed a behavior similar to single wMWCNTs in all the conducted tests. The measured effects of TCC on Chl-*a* and POC concentrations concerning the benthic biofilm contribute only to a very low extent to the overall effect. The major part has to be assigned entirely to the presence of wMWCNTs, which resulted in a decrease in Chl-*a* concentration and an increase in POC concentration. The significant decrease in Chl-*a* concentration is attributable to the wMWCNTs. Shadowing effects may provide a potential explanation. Yet, the effect induced by the complex is even slightly reduced compared to wMWCNTs alone. It is possible that the scattering of the mixture TCC + wMWCNTs can be explained by the fact that the wMWCNTs clumped more with the TCC and thus did not cover the algae over a wide area, producing selective shading. This can also be explained by the fact that the scatter among the individual wMWCNTs is not so large, but the Chl-*a* concentration is still significantly different from all other analyses, except for TCC + wMWCNTs. Furthermore, the attempt to bind TCC to the wMWCNTs was successful reaching 80% bound TCC. For irradiated CNTs, this approach had already been successfully applied (Ion et al. 2019). In the present study, it could be demonstrated to work equally well with weathered carbon nanotubes. The TCC concentration measured at baseline decreased to levels below the LOD within the 5 days of exposure, as the desorption process occurred both in the tests with TCC as a single compound and in the tests with complexes of coupled TCC + wMWCNTs. With regard to the tested single substances TCC and wMWCNTs as well as the TCC + wMWCNTs, further research approaches should be aimed at. These should have a strong focus on the respective uptake pathways of the different organisms. In addition, several higher organisms could be studied together in one exposure vessel to potentially draw more accurate conclusions.

## Conclusion

In the present work, we review and analyze various aspects regarding the contrasting results in the literature about toxicities and effects of TCC on aquatic organisms. Previously reported findings consistently indicate a high toxicity of TCC when investigated in single species experiments. Here, in contrast, we exposed TCC to complex communities of aquatic organisms, and our results suggest that the toxicity effects are clearly reduced. We attribute this finding to synergistic effects among the cohabiting organisms strengthening their resistance against potential pollutants. The varying data from the literature on TCC also mean that it is very difficult to categorize this substance in terms of its actual toxicity. Quite crucially, TCC is extremely adsorptive and only poorly soluble in water. Therefore, TCC might not be present in surface waters for long periods in significant concentrations and, accordingly, it may be difficult to detect. Thus, TCC is always striving to find a binding partner to which it can attach. Some literature describes TCC concentrations in surface waters and most studies are on aquatic organisms, such as filter feeders. However, we believe it is much more critical to look at the community of benthic organisms, as this is much more reflective on the properties of this chemical. In order to get more accurate information about the toxicity of TCC, it is very important to use such aquatic organisms, which are also expected to be the most exposed in the environment. In our short-term study, we showed that grazer organisms are not very sensitive to TCC. The discrepancy between the reported effects of this substance toward organisms, ranging from acutely toxic at low concentrations to no mortality, is very large. In addition, we here also studied corresponding toxicity effects when applying TCC together with wMWCNTs to complex aquatic communities. This coupling test revealed that among the substances TCC and wMWCNTs, there is no synergistic effect. Rather, the wMWCNTs alone are primarily responsible for the toxicity. Furthermore, the coupling test shows an abrogating effect of the wMWCNTs by the TCC, which was demonstrated by comparing the Chl-*a* concentrations. Contrary to our expectations, any significant effect is attributable exclusively to the wMWCNTs. Nevertheless, much research is still needed, as this chemical (TCC) is very difficult to detect, resulting in different assumptions from the literature. The degradation behavior of TCC should also be mentioned here, which can be tested in long-term studies in order to draw absolute conclusions regarding its toxicity.

## Data Availability

On inquiry, the data presented in this study is available from the authors.
